# Genito-Urinary and Syphilitic Diseases

**Published:** 1900-09

**Authors:** Byron H. Daggett

**Affiliations:** Buffalo, N. Y.


### GENITO-URINARY AND SYPHILITIC DISEASES.

CONDUCTED BY BYRON H. DAGGETT, M. D., Buffalo, N. Y.

### THE SIGNS OF INHERITED SYPHILIS.

DAWBARN, before the New York Academy of Medicine and printed in the November number of the *American Journal of Dermatology and Genito-Urinary Diseases*, said: The mortality of children of syphilitic parents is frightful and this inheritance is frequently met in the practice of men of all specialties. Fournier, after a careful examination of statistics, states that only one child in four survived. The Moscow test of 2,000 cases gives about the same percentage. A large majority died the first year. In forty cases of acquired syphilis all recovered but one. A prompt recognition of the presence of congenital syphilis is essential. Hence a compact form of the clinical signs is important. Twenty-seven diagnostic points are herewith presented. They are not all of equal pathogenic value, but are all sustained by high authority. The symptoms and their diagnostic value will be briefly considered. There is no chronological order of involvement of this congenital disease and its symptoms will be diagnosticated as those which are early and those appearing later. The initial lesion of inherited syphilis will not be considered. (Quoting Diday and Smith):

1. "Next to the look of little old men, the most characteristic sign is the sallow color of the skin." The newborn are rarely plump, but are wrinkled like an old man, still they may be born plump and shortly become syphilitic.

2. The umbilical cord is extremely thickened and appears swollen; is apt to be very long and is slowly detached from the navel. This hypertrophy is due to a partial endophlebitis obliterans, occasionally noticed, and involves the umbilical vein.

3. Pemphigus syphiliticus—mortality almost 100 per cent. My first case was diagnosticated by my preceptor, and it died as he had predicted. The vesicular syphilide is not so fatal, but it is a very severe type of infection. In the inherited type only do we find bullæ or vesicles.

4. The common eruption is most frequently an erythema. The nodules appear upon the abdomen and genitals, pinkish at first and later coppery in color. The rash appears about three weeks after birth. The size of the spots after development is about that of the baby's finger nails. Subsequently these syphilides become polymorphosis and more irregular than those of acquired syphilis. In about 25 per cent. of inherited syphilis only do we find skin eruptions.

5. *Condylomata* often appear early and locate about the mucocutaneous junctions—commonly about the anus.

6. "*Snuffles*" is a common sign and is exceedingly suspicious.

7. Mucous patches, ulceration and later gumma about the larynx cause hoarseness.

8. Mucous patches are common and may be found upon any of the mucous surfaces.

9. Stomatitis and pharyngitis are almost constant phenomena, ulceration appearing later.

10. Enlargement of the lymphatics is not always present in the congenital type.

11. Hemorrhages and ecchymotic spots have diagnostic value. Hemorrhage from ulcerated or apparently sound mucous membranes.

12. Virchow's "white pneumonia," diagnosticated post mortem and is due to fatty degeneration of the vesicular epithelium. This condition is found in still-births and those dying soon after birth. In older cases it is interstitial pneumonia or gumma. Pneumonic signs at or soon after birth are suspicious.

13. Involvement of the liver is frequent, such as interstitial hepatitis, gummata or amyloid degeneration. Jaundice is never present.

14. Enlargement of the spleen occurs in a majority, if not in all cases and has great diagnostic value.

15. Certain eye symptoms are diagnostic—"ground glass cornea," keratitis, iritis, choroditis, retinitis and neuritis may also be found. Keratitis is the one of greatest diagnostic value.

16. Inflammation of the middle-ear, when associated with other symptoms is important as a diagnostic symptom.

Hutchinson's "triad of symptoms" are: notched teeth, ground-glass cornea, and otitis media.

17. Alopecia suggest syphilis.

18. Painless orchitis is rare, but a valuable sign.

19. Neuroses, although infrequent and occur late, yet they require careful examination; chorea, epilepsy and paralysis.

20. Osteochondritis, involving the shafts and epiphyseal junctions of the long bones is among the commonest and earliest sign.

21. Later and even more characteristic is periostitis. "Nodes of Parrot" is a notable manifestation of syphilitic periostitis.

22. Symmetrical and multiple dactylitis syphilitica.

23. Claw form of the finger nails.

24. Early appearance of the teeth, with bad color and tendency to decay. To be born with teeth is bad luck.

25. Hutchinson's teeth. Notching of the upper central incisors, peg and screw driver shapes. Found only in the permanent teeth and not always present.

26. Irregularities of bone development, found in the later stages, hypertrophies, asymmetries and necrosies, pug and saddle-back noses, and saber shape tibia.

27. Stigmata.

